# Labour pain experiences and perceptions: a qualitative study among post-partum women in Ghana

**DOI:** 10.1186/s12884-017-1248-1

**Published:** 2017-02-22

**Authors:** Lydia Aziato, Angela Kwartemaa Acheampong, Kitimdow Lazarus Umoar

**Affiliations:** 1 Department of Adult Health, School of Nursing, College of Health Sciences, University of Ghana, P.O. Box LG 43, Legon Accra, Ghana; 2 School of Nursing, Wisconsin International University College, Accra, Ghana; 3 Department of Adult Health, School of Nursing, University of Ghana, Legon Accra, Ghana

**Keywords:** Ghana, Labour pain, Pain experience, Pain, Qualitative research

## Abstract

**Background:**

Women have experienced severe labour pain over the years and various attempts have been made to effectively manage labour pain. However, there is paucity of literature on the labour pain experience and perceptions about labour pain with the contemporary Ghanaian health system. Therefore this study sought to gain an in-depth understanding on labour pain experiences and perceptions of post-partum women.

**Methods:**

The study adopted an exploratory descriptive qualitative approach and collected data through individual interviews. Informed consent was obtained from all the participants who were purposively sampled. After transcription of interviews, the data were analyzed inductively using content analysis techniques.

**Results:**

Women in this study experienced pain during labour rated as mild, moderate and severe and the pain was felt at the waist area, vagina, lower abdomen and the general body. The women expressed labour pain through crying, screaming and shouting. They prayed to God to help reduce the severe pain. Some women endured the pain, cried inwardly and others showed no sign of pain. Some women believed that crying during labour is a sign of weakness. Pain reliefs such as pethidine (Meperidine) was occasionally given. Non-pharmacologic measures employed included walking around, deep breathing, side-lying, waist holding, squatting, taking a shower and chewing gum. The individuality of pain experience and expression was emphasized and the socio-cultural orientation of women made some of them stoic.

**Conclusion:**

We concluded that it is necessary for all health professionals to manage labour pain effectively taking the socio-cultural context into consideration.

## Background

Most women perceive labour pain and childbirth as most severe and agonising event of a woman’s existence [1, 2]. Childbirth is a physiologic and natural process that has been undertaken by women over the years with professional assistance [3, 4]. Although there are no underlying pathological processes, labour is linked with a painful experience [5], so a lot of women are worried about labour pain and how they can be relieved of pain [6, 7]. The women’s experiences with labour make them anticipate the pain with intense worries, panics, and depression, mostly when the progression of labour is delayed [8, 9]. Baker et al. [5] found that women classified labour pain intensity as mild, moderate or severe, with increasing intensity from the onset of contractions to complete dilatation of the cervix. At the commencement of labour, the pain occurs intermittently manifesting as abdominal aches [10], pelvic pain [11] and backache [12]. One thing that women recall about labour is high intensity pain [13].

When labour pain intensifies, the women express pain through crying and screaming [14, 15]. Such expressions help women in labour to cope with their pain [16, 17]. On few occasions, some women disregard labour pain and are stoic [18, 19]. Obuna & Umeora [20] revealed in Nigeria that ignorance of existing pain relief by women and cultural prejudice accounts for inadequate demand for labour pain relief. Among some Ghanaian cultural groups, there is a belief that, it is humiliating if others know one cannot bear labour pain [21]. As a result, women who are unable to endure labour pain are labelled as emotionally weak [22].

Pharmacological agents such as pethidine (Meperidine) [23] and other analgesics are used to effectively control labour pain [24]. In addition, the use of herbal medicine has been reported to relieve labour pain [25]. Previous studies also report the use of sterile water injection to control labour pain [26]. It is distressing to note that some women are taught not to use pain medications but to endure labour pain as a symbol of womanhood [27], and a sense of pride [28, 29]. This shows an insufficient antenatal education of women on the management of labour pain [30]. In addition, non-pharmacological approaches such as emotional support to women from their partners [31], family members, professional or non- professional staff help women to control their labour pain [2]. Some women employ other non- pharmacological pain relief measures during labour such as breathing exercises, taking showers [3, 6, 23], assuming specific positions and moving about [3, 6, 32] to control their pain. Women also cope with labour pain through prayer [19, 33] for God to reduce their pain [21].

The need for effective labour pain management cannot be overemphasized since labour pain makes the woman tired and this could affect her ability to bear down during the second stage of labour [34]. Psychological reactions such as anxiety or apprehension results from severe labour pain which in turn leaves a negative impact on the psychological experiences of women in labour and effective pain control has a high tendency of relieving the effects [2]. Therefore both pharmacological and non-pharmacological approaches are necessary to relief labour pain effectively [13]. In Ghana however, support persons such as husbands and family members are not allowed into labour wards due to lack of privacy [22]. Midwives play major supportive roles in alleviating labour pain [33] especially in instances of positive interaction with women in labour [35] by showing caring and encouraging attitudes to women experiencing labour pain [8].

Women’s perceptions and experience of labour pain and how they cope with pain have been explored [22], but there is still limited literature on the phenomenon of labour pain experiences of post-partum women in a Ghanaian context. This study seeks to address this gap in the literature. An understanding of the unique experiences of women in labour within the socio-cultural context of Ghana could inform care interventions for labour pain. This rests on the premise that pain is an individual phenomenon and socialization influences pain behaviour and pain management [36]. Hence this study sought to gain in-depth understanding of the experiences of labour pain and perceptions about labour pain.

## Methods

### Study design

An exploratory descriptive qualitative approach was used to gain an in-depth understanding of the labour pain experiences and perceptions of women in this study since much is not known about the phenomenon in Ghana. The approach was appropriate because it allows the participant to fully describe experiences and perceptions and also permits the researcher to probe emerging themes [37]. Thus, the labour pain experiences of women and their pain perceptions were fully explored and described in this study.

### Study location

The study was conducted at a tertiary health facility in Accra, Ghana. The participants were drawn from the post-natal clinic where post-partum women sought day care services. The women lived within the Accra Metropolis and were not on admission and their babies were also healthy. The context of the study attracts women from various parts of Ghana and this provided the opportunity to explore the experiences and perceptions of women from all the geographical areas in Ghana. Ghana has many ethnic groups and women may be socialized differently in pain expression and behaviour. As a lower middle income country, there is resource limitation within the clinical setting and this could hinder pain management [38]. Within the context of the study, labour pain and its management are not regularly discussed at the antenatal clinics. High workload and inadequate midwives reported in a previous study may contribute to this gap in care [39].

### Sampling and data collection procedures

Fourteen participants were purposively sampled and were engaged in individual interviews. The interviews were conducted in English, Twi, Ga and Ewe and the first author who is fluent in these languages conducted all the interviews. A semi-structured interview guide was used to conduct the interviews. Open ended questions were used to generate responses and these were probed until a full understanding was achieved. For example participants were asked ‘*please tell me what you went through during your recent childbirth’*; ‘*please describe how you were supported during the labour pain’*. The first author is an experienced qualitative researcher and used appropriate techniques during the interviews to generate data in this study. Thus, leading questions were not asked which ensured that the participants were allowed to express themselves freely. There were no new findings after the thirteenth participant. Each interview lasted between 30 and 45 min. The venue and time of the interviews were at the convenience of the participants. All the interviews were audio recorded and later transcribed verbatim.

### Data analysis

Data analysis occurred concurrently in this study following the techniques of content analysis described by Burnard [40]. However, because the NVivo software was used to manage the data, manual processes described were not employed. The transcripts were read several times to gain a full sense of the participants’ world. The transcripts were coded by reading line by line and assigning a word or phrase that accurately capture the essence of the datum read. Through this process, some of the codes generated such as facial expression, crying, shouting etc. were grouped under pain expression. Similar codes were grouped, re-grouped and refined to generate themes which were meaningful units on labour pain experiences and perceptions of women. The research team discussed the themes to ensure that the data were faithfully represented. The data were subsequently exported into the NVivo software version 11 and this was used to manage the data. The study held that the lived labour pain experiences of post-partum women were reported during interviews conducted in a non-threatening environment and faithfully represented their world.

### Trustworthiness of the study

A number of processes were used to ensure the trustworthiness of this study. Concurrent analysis ensured that emerging themes were probed in subsequent interviews to gain full understanding of themes. The same interview guide was used for all the women. Interviews that were conducted in Twi, Ga and Ewe were discussed with experts in these languages to ensure accuracy of the transcripts. The research team discussed the themes generated and ensured that no aspect of the data was left out. Detailed field notes were kept which allowed verification of findings and study processes. Direct verbatim quotes were used to support the findings and this gave voice to the women in this study.

### Ethical considerations

Ethical clearance was obtained from the Institutional Review Board of the Noguchi Memorial Institute of Medical Research of the University of Ghana. All the participants gave informed consent and were given the opportunity to withdraw from the study without any repercussions. Identification codes were used to represent the participants and these were used to present findings. The data presented contains no identifying information. The researchers avoided prejudices and remained non-judgmental throughout the process of data collection and analysis. Permission was sought to record interviews and publish data generated.

## Results

### Participants’ background

The participants were made up of 14 post-partum women who had delivered within two months. They were aged between 18 and 35 years and they were all married except one participant who was single. Thirteen participants were Christians and one was a Muslim. All the participants had a normal vaginal delivery except one who had a caesarian section on account of multiple pregnancies.

The findings were described on themes such as: Labour pain location and intensity, Labour Pain Expression, Perceptions of women on labour pain expression, Experiences related to labour pain relief measures, Experiences of women on support from family and midwives during labour pain and Women’s experience of negative attitudes of midwives.

### Labour pain location and intensity

This theme describes the location and intensity of labour pain women experienced. Women in labour felt pain at different locations. Some participants felt labour pain in their waist and vagina with the urge to defaecate and urinate. Other women experienced bodily and lower abdominal pain during labour.

“…*the pain was at the waist area, it was really painful like something pushing at you*” (PP12); “*I felt pains in my vagina and my waist I felt like going to the toilet and urinating*” (PP2). “*The pain was all over my body; … I also felt the pains in my lower abdomen*” (PP8).

The degree of pain experienced by the participants was rated as moderate and severe. However, a few of the women experienced pain that was described as *bearable* and it lasted for a short period during labour.

“*Oh I did not really experience much pain; I was able to bear it”* (PP8); “*…my experience was very short”* (PP9-11).

Some women had moderate labour pain which occurred intermittently and described the pain as sharp and hurting. When the pain worsened, they felt like ‘*being torn apart’* or *‘broken into pieces’* and they could neither sleep nor withstand the pain.

“*It got to a point the pain reduced but after a while it was severe”* (PP9).

“*I was suffering a lot … I felt a sharp pain and when it increases, it is like you are being torn apart. That point when you feel like you are being broken into pieces”* (PP2)

*“…I was hurting; then it was getting worse and worse so I couldn’t sleep anymore …I was in real pain”* (PP12).

A few women could not lift their leg, hold or do anything due to severe labour pain but when pain subsided, they regained their movement. Some women stated that labour pain was severe during the evening and at night than during the day.

“*… When I wanted to lift my leg, I couldn’t; the pain was so severe… It took about 15 min before I was able to move again.”* (PP14)*; “I could not hold anything; …I could not do anything”* (PP6).

*“I experienced the severe pains in the evening; …it was not severe during the day”* (PP8); *“…labour started in the night…I was having severe labour pains; it was really painful*” (PP12).

### Labour pain expression

Some of the women in this study cried openly and called on God to deliver them from the unbearable pain. However, other participants said they cried silently and shed tears without their conscious awareness. Therefore ‘crying’ during labour in this study was not always associated with sound.

“*During the pain, I was calling on God because the pain was unbearable. …I was crying”* (PP12). *“I was crying”* (PP7).

“*I was not crying out loud but I was in pain and was crying inwardly …”* (PP6); “*I shed tears throughout silently without crying out loud …the tears kept flowing even when I was talking*” (PP11).

Some women shouted and were agitated when in pain such that they scattered items in their rooms and removed their clothing. The shouting was more pronounced during the second stage and when labour was severe.

*“…I shouted throughout when the pain was very severe and during the second stage till I delivered” (PP8); “I never imagined I will behave that way; I was shouting…oh God I can’t…I scattered all the things in the room, the midwives would come and cover me up and I would remove everything”* (PP12).

Some women shouted ‘*Jesus, Jesus, Jesus; God help me’* till they gave birth. They felt that the labour pain was more painful than menstrual pain. The shouting was continued until they gave birth.

“*I was shouting Jesus, Jesus, Jesus; that was the only word I was using and I shouted ‘God help me!’ several times because although I had menstrual pain, the labour pain was more severe; I never stopped shouting till I delivered”* (PP9).

There were participants who experienced labour pain but concealed it or showed no sign of pain. Due to the severe labour pain, the women were happy after birth because labour pain was over.

*“I was lying down showing no signs of pain; I just kept the pain in me without shouting” (PP5); “I just harboured the pain and it was not obvious to other people”* (PP8).

*“Oh! I felt happy that at least the pain was gone and I had my baby too”* (PP9).

### Perceptions of women on labour pain expression

Women in this study perceived labour pain as normal. Some were of the view that they were to be quiet and not allow others to realize they were in pain. Hence some participants were advised not to allow other people notice that they were experiencing labour pain. A participant considered labour pain expression *funny* and expected women to bear the pain without any *obvious* signs.

*“… my mum advised me that I should not let anyone notice my pain …when I got to the hospital, I remembered what my mother told me so I was silent* (PP3);

*“…it is funny to me when I see people exhibiting signs of pains when they are in labour … Some people would be screaming and asking for the nurse’s help whiles the nurse too cannot go through the pain for you. Yes, it is painful but you can go through without making it obvious as I did”* (PP8).

Some participants from the northern part of Ghana perceived that women who cried during labour were weak. As a result, they were stoic during labour pain so that after child birth they could boast that they endured labour pain without crying. Those from the south were perceived to express more pain. The women from the south also held the same general perception in this study; although individual differences were reported.

“*…in the north, the women who cry during labour are tagged as weak women …you have to bear the pain so I cannot allow myself to cry so that after the baby comes out, I can also boast that I was able to go through labour without crying …those who are not from the north cry and shout”* (PP10)

*“…those of us from the South make a lot of noise when in labour than those from the North but it depends on the individual”* (PP8)

Some women perceived that screaming during labour pain led to exhaustion and depleted the energy required for pushing leading to episiotomy during childbirth.

“*…if you do not scream during the pain, it helps you to save the energy so that when it is time you can push but if you scream, you would be exhausted when the time comes for you to push and you might end up with episiotomy”* (PP8).

Some women perceived labour to be painful before they went into labour and that made them *scared*. A participant who had triplets was afraid because she was told she may die when she is sent to the theatre and that carrying triplets was *‘strange’* with impossible spontaneous vaginal delivery.

“*I was scared, I was told labour was painful and I really experienced it …it is really painful”* (PP12).

*“…they said if I go to the theatre I will die; …when I told my mum I was carrying triplets, she said it’s strange and that I would not be able to give birth myself unless I was operated. I was afraid”* (PP13).

### Experiences related to labour pain relief measures

During painful contractions, some women paced continually and rested when the pain subsided; because, lying down still aggravated their pain. Some women experienced pain relief from lying on the side, holding the waist, squatting and praying.

“*… I just walked up and down during labour because when I lay down, it was painful so I sat a little or lay a little; but, when the pain started I had to walk around and it helped to bring the pain down”* (PP3).

“*… I have previous knowledge that when you are in pain, you should lie on your side and when I did that, it helped” (PP12); “I squatted and it helped bring the pain down and I also prayed”* (PP12); “*Sometimes I placed my hand around my waist to relieve the pain”* (PP10).

Other participants who had knowledge in deep breathing exercises breathed through the mouth during painful contractions. A participant experienced relief of labour pain when she chewed gum and spent some time under the shower as these helped to take her mind off the pain. However, a woman was pre-occupied with the thoughts of the pain and how to relief it.

*“I have previous knowledge that when you are in pain and you breathe through your mouth. When the contractions start, I was doing it and it helped reduce the pain” (PP12).*

*“I just chewed gum when the pain intensified… I spent some time under the shower and these helped to reduce the pain” (PP8).*

*“…the only thing I was thinking about was the pain and how to relieve it; … what to do to let the pain go away” (PP9).*

Some of the women experienced pain relief when they were given analgesics such as pethidine (Meperidine) injection which wore off after a short while. A participant experienced the administration of *placebo* (giving other drugs other than analgesics when in pain) and she slept after the *placebo* was given. In addition, two participants who gave birth with a traditional birth attendant experienced pain relief when they chewed guava leaves and used herbal enema.

“*…if they give me the pethidine within some few minutes or hours it wears off”* (PP11). “*…I chewed guava leaves and the pain was better”* (PP5).

“*…they gave me something in the infusion to relieve my pain but I knew it was a placebo; “…but when they put it in, I fell asleep”* (PP8).

“*…I used herbal preparation as enema weekly and when I went into labour, the pain was bearable”* (PP2).

### Experiences of women on support from family and midwives during labour pain

Some participants received support from midwives. They noted that the midwives were *nice, patient, pampering* and encouraging during labour pain.

“*I was pampered by the midwife … I mean she was nice to me, she gave me the best attention”* (PP12); *“… one of the midwives told me to open my legs but because of the pain I could not open and she told me to take my time. She was patient with me.”* (PP6); “*The midwife said I should be calm and she encouraged me to push”* (PP7).

Some of the participants were prepared psychologically by their mothers towards labour pain and to avoid pushing if they had no urge.

*“… my mum always said labour pain is even worse than menstrual pain so she always prepared my mind towards the pain I will go through, so I was ready”* (PP8); *“… my mum said if I don’t have the urge, I shouldn’t push even if the pain is severe”* (PP13).

The participants were also told by their mothers that labour pain was a natural ordeal women are expected to experience as instituted by God and this contributed to the psychological preparation to endure the severe labour pain.

*“…our mothers already prepared us to go through severe pains so that you know what your mother went through. …that is how God created us women and made us to go through pain during labour. So we have to feel the pain during labour so it’s worth it”* (PP9).

Some women received encouragement and empathy from their aunts and husbands that helped them cope with labour pain.

“*…so my aunt really helped me by encouraging me” (PP8); “…my husband he will say, ‘sorry, sorry, it will soon be over’ you can see that he feels for me”* (PP3).

### Women’s experience of negative attitudes of midwives

Some of the participants in this study reported some negative attitudes of midwives during labour pain. Others bemoaned that the midwives neither showed empathy, consoled nor reassured them but, they saw them as a nuisance when they expressed labour pain.

“*…sometimes the way they behave is as if they haven’t gone through labour before because when you are telling them you are in pain and expecting them to console you, they don’t mind you. So, it is as if you are disturbing them”* (PP8).

Some of the women were shouted on by midwives to keep quiet when they complained that they were in pain. Other women were only told to limit the noise when they expressed labour pain through shouting. However, some were encouraged to persevere throughout labour pain rather than being given medication to relieve the pain.

“*…they were shouting on us… the midwife would ask you to be quiet because you are not the only one around and you are not even close to delivery”* (PP8).

“*…if the midwife realize you are making too much noise, she will just come and tell you, madam the noise is too much so don’t shout”* (PP9).

“*…the midwife will rather encourage you to go through the pain …instead of giving some injection to take the pain away*” (PP11).

## Discussion

This study described the labour pain experiences and perceptions of post-partum women in Accra, Ghana which highlighted the individuality of pain experience and perceptions. The perception that labour pain is most severe and agonizing as reported by women in this study resonate Costa-Martins et al. [1] and Wong [2] finding about labour pain as very severe. Women in this study reported mild, moderate and severe labour pain. Researchers’ continuous report of labour pain pre-supposes that labour pain is poorly managed within different clinical settings. Ghanaian women go through labour expecting painful experiences. This finding is congruent with previous studies in other countries where labour pain is anticipated [7, 41].

Labour pain expectations could be attributed to previous information on labour pain and personal or other people’s previous experiences with labour [4, 42]. This, therefore calls for effective education on labour pain relieving modalities [43], especially with reports of insufficient antenatal education of women on the management of labour pain [22, 41]. Education on labour pain management should be introduced early and regularly re-visited until delivery so that women would understand pain management approaches used. Also, the women considered labour pain as a natural phenomenon women are expected to experience as instituted by God. This perspective is similar to perspectives of midwives in Ghana reported in a previous study [39]. Mostly, the view of labour as a normal inevitable process helps women to cope better with the pain [44]. This does not mean that labour pain management should not be given much attention because even in a normal physiology of labour, women still experience pain [43].

The expression of labour pain among women was individualistic [45] and exhibited as crying, screaming and shouting [14, 46]. Women who reported that they felt like being torn apart or broken into pieces during severe labour pain have higher tendencies of reporting negative experiences of labour, compared to those with mild labour pain experiences. Although pain intensity is not assessed routinely in the Ghanaian clinical setting, the intensity of pain can be assessed with the use of validated pain assessment scales and the findings can guide pain management decisions [47]. It is noted that childbirth experiences are immensely influenced by the experience of women with pain and the support from healthcare team members [48]. The labour pain the women felt was located in the abdomen, waist, vaginal and the entire body. In other studies, labour pain was reported at the pelvic girdle [11], the back [12] and the abdomen [10]. The different locations further shows the subjectivity of pain. Therefore it is crucial that midwives recognize the individuality of women in respect to labour pain and manage them as such.

The severe pain made some women call Jesus or God for help [16, 19, 49]. Praying to God for help is a demonstration of spirituality or faith during labour that God can ameliorate labour pain as well as have a safe childbirth [21, 50]. Some women also coped with labour pain because they considered labour pain to be normal during childbirth and it should be endured without shouting as supported by Ampofo and Caine [22] that crying during labour pain is a sign of emotional weakness. Other authors also reiterate that enduring labour pain without pain reliefs is a symbol of womanhood [27] and a source of power and pride for coping with labour pain [51]. These beliefs among women may be fueled by their ignorance of pain relief approaches during labour [20]. Stoicism during labour suggests that women may experience labour pain without verbalizing, expressing labour pain and could abhor pain reliefs just to preserve their prestige. This calls on health professional to always take reliable socio-cultural history of pregnant women so as to anticipate their pre-labour perceptions.

In this study, intramuscular pethidine injection was administered to relieve pain only when pain was very severe. This finding indicates that labour pain management is based on health professional’s discretions such as maternal suffering [13]. In another finding, it was reported that pethidine has a short-acting effect as confirmed in this study [8]. Health professionals must therefore not forget to administer the next dose of short-acting analgesics during labour. Women in this study also used non-pharmacologic pain relief measures such as breathing exercises, assuming side-lying positions, waist holding, squatting, taking showers, chewing gums and walking up and down to manage and cope with labour pain as reported in previous studies [3, 6, 23, 32]. This suggests that health professionals must provide opportunity in the labour ward for the women to utilize their chosen methods during labour pain.

Also in this study, women who gave birth through the help of traditional birth attendants reported that they were not given any analgesics but rather chewed guava leaves and used herbal enema to relieve labour pain. This resonates the finding of a previous study about the traditional practices such as the use of herbal preparations during childbirth [25]. However, the efficacy of the herbal preparations in the management of labour pain was not investigated in this study. Other studies report that herbs are considered to be harmless with no adverse effects as compared to drugs [52, 53]. Women could be allowed the opportunity to choose either herbal medicine or western medicine [54] when in labour to alleviate pain.

This study found that support persons during labour were midwives, spouses and other family members. Some midwives played a major role during labour pain such as caring and encouraging the women during labour pain and the women were glad for the care received. However, some of the women in labour encountered negative midwives’ attitudes such as shouting and lack of empathy as supported in another study [5]. This behaviour may deter pregnant women from seeking professional care during child birth [55]. There is therefore the need for midwives to receive continuous in-service training on labour pain management and communication skills to enhance their care of women in labour.

This study included only one Muslim woman and this could limit the application of the findings to Muslim communities. Only post-partum women were involved in the study and some of them may not recall all their pain experiences during labour. However, the strength of this research is that it was able to gain an in-depth understanding of the labour pain experiences and perceptions of women through an inductive exploratory approach.

## Conclusion

This study gained in-depth understanding of the women’s experiences of labour pain and their perceptions on it which showed the individuality of labour pain experience. Women continue to experience labour pain and the findings re-enforce the individuality of pain. The notion that labour pain is natural and must be endured should be de-emphasized during health education programmes because it is the right of every woman to have adequate pain relief during labour. Midwives should support and encourage women during labour and allay their fears especially when the need for caesarian section arises. A good relationship between the midwife and the woman in labour is advocated so that women will seek professional care which could reduce preventable complications during childbirth. The cultural background of the woman in labour must be taken into consideration because some are socialized to be stoic. Therefore labour pain must be assessed adequately to inform effective pain management.
